# Simple analytical solutions for the optical properties of multilayered confocal prolate spheroids in the quasistatic approximation

**DOI:** 10.1186/s11671-026-04607-5

**Published:** 2026-05-27

**Authors:** Mugahid Ali, Fumin Huang

**Affiliations:** 1 Centre for Doctoral Training in Integrated Photonics and Advanced Data Storage (PIADS), School of Mathematics and Physics, Queen’s University Belfast, Belfast, BT7 1NN UK; 2 Present address: School of Physics, CRANN, AMBER and CONNECT, Trinity College Dublin, Dublin2, D02 PN40 Ireland; 3 Centre for Quantum Materials and Technologies, School of Mathematics and Physics, Queen’s University Belfast, Belfast, BT7 1NN UK

**Keywords:** Optical properties, Plasmonics, Quasistatic approximation, Core-shell, Multilayer spheroid, Multilayer sphere

## Abstract

We present simple analytical solutions to Laplace’s equation for multilayered confocal prolate spheroids within the quasi-static approximation, tailored to configurations where the polarisation of the incident field aligns with the spheroid’s major axis. The field potential in each region is expressed as a superposition of two analytic functions, with amplitude coefficients conveniently determined through a compact matrix formulation derived from the boundary conditions. A key advancement of this work is the derivation of an explicit expression for the amplitude coefficient $K_0$ of the perturbed field at the surface of the multilayered spheroid, obtained from the ratio of two matrix determinants. This coefficient inherently incorporates all constituent material properties and geometrical parameters of the nanostructure. Building on this, we derive a simple and general expression for the effective polarisability $\alpha$ of the multilayered spheroidal nanoparticle, which serves as the foundation for calculating its optical characteristics, including scattering and absorption. In contrast to previous models, the proposed analytical expressions enable rapid and accurate evaluation of optical responses–such as scattering, absorption, field enhancement, and plasmonic resonance–across arbitrary multilayered spheroidal configurations. Numerical examples of two-, three-, and five-layer structures are provided to demonstrate the model’s accuracy and computational efficiency, underscoring its potential as a powerful analytical tool for the design and optimisation of complex plasmonic nanostructures.

**Supplementary Information:**

The online version contains supplementary material available at 10.1186/s11671-026-04607-5.

## Introduction

The geometry of the spheroid is crucially important in nanophotonics. Nonspherical nanoparticles are commonly represented as spheroids; for instance, a nanorod is typically modelled as a prolate spheroid [1], while a nanodisk or short cylinder can be modelled as oblate spheroids [2]. Such approaches have been widely adopted across various research areas to investigate the scattering problems of electromagnetic waves. For instance, in surface-enhanced Raman spectroscopy (SERS), scatterers are often modelled as spheroids [3]; in scanning near-field optical microscopy (SNOM), the tip is approximated as a prolate spheroid [4]; in biophysics, cell membranes are often depicted as a coated spheroid [5, 6]; and in atmospheric physics, raindrops are modelled as spheroids [7]. While homogeneous spheroidal particles are of interest in many applications, stratified spheroids consisting of multilayered materials of varying optical properties offer distinct advantages. These particles can produce a broad range of optical properties not attainable in homogeneous single-layer spheroids, owing to the flexibility in choosing the eccentricity, number of layers, thickness and constituent materials. This versatility enables active tuning and optimisation of the optical properties of nanoparticles, facilitating the design of complex optical properties to empower specific applications. Therefore, investigating the optical properties of multilayered spheroids is of significant importance in nanophotonics.

The scattering theory for a homogeneous spheroid was first developed by Asano and Yamamoto [8]. This foundational work was subsequently extended to multilayered spheroids by several authors, including Farafonov [9, 10] and Gurwich et al. [11]. However, these analytical solutions are intricate, as they are expressed through expansions in a series of spheroidal functions. Calculating the expansion coefficients and numerically evaluating the spheroidal functions is nontrivial, necessitating substantial programming efforts and computational resources, which limits their practicality.

A more general treatment of multilayered ellipsoidal inclusions was proposed by Sihvola et al. [12], who introduced a transmission-line analogy in which the fields within the layers and the surrounding medium are represented as inward-propagating and reflected components, resulting in a dipole-type field in each region. The field amplitude coefficients across interfaces were then obtained through a cascade of matrices that describe both the forward- and backwards-propagating fields. Although comprehensive, this formulation involves multiple matrix operations that become increasingly cumbersome with the number of layers. Moreover, the model was originally developed to investigate microwave attenuation in melting hailstones and has not been applied to optical or plasmonic systems.

Many nanophotonic applications, however, concern nanoparticles that are much smaller than the wavelength of light, allowing the quasi-static approximation to simplify analytical solutions considerably. In this regime, a simpler spheroidal model provides sufficient accuracy while retaining analytical clarity and computational efficiency. Aligning the polarisation of the incident light with the nanoparticle’s major axis further enhances plasmonic resonance effects. Importantly, this configuration does not restrict the generality of ellipsoidal scatterers. Spheroidal nanoparticles–characterised by two equal minor axes–constitute a special case of ellipsoidal particles; aligning the incident field along the principal axis simplifies the analysis. For other oblique orientations, the incident field can be decomposed into components along the principal axes, and the overall scattering response arises as the superposition of these orthogonal contributions. This approach is widely adopted in nanophotonics and underpins many plasmonic phenomena and applications.

Building on these considerations, we develop a simple and fully analytical model for the scattering of multilayered spheroids within the quasi-static approximation. The electric potential in each region–core, shells, and surrounding medium–is represented as a superposition of two analytic functions. The field amplitude coefficients are obtained from a compact set of simultaneous linear equations derived directly from the boundary conditions at each interface. The overall optical response is determined solely from the perturbed field at the surface of the nanostructure, whose amplitude coefficient, $K_0$, is expressed as the ratio of two matrix determinants. The effective polarisability $\alpha$, which governs the scattering and absorption cross-sections, follows in closed form and depends exclusively on this coefficient. This formulation eliminates the need for cascading matrix operations, providing a numerically stable and computationally efficient framework for evaluating the optical spectra and near-field distributions of multilayered spheroids.

The simple analytical solutions provided by our model allow for swift evaluation of a range of optical properties (scattering, absorption, field distribution, field enhancement, plasmonic resonance, etc.) of multilayered spheroids, which can be easily implemented on standard desktops/laptops without resorting to extensive programming efforts and computing resources. The model is applicable to spheroids of an arbitrary number of layers. To demonstrate the capability and efficiency of the proposed model, we provide numerical results of the optical properties of spherical and spheroidal nanoparticles of 2, 3, and 5 layers; additional examples of a 4-layer spherical structure are provided in the Supplementary Information (Fig. S4).

## Mathematical formulation

### Solutions of laplace equation for a homogeneous prolate spheroid

**Fig. 1:**
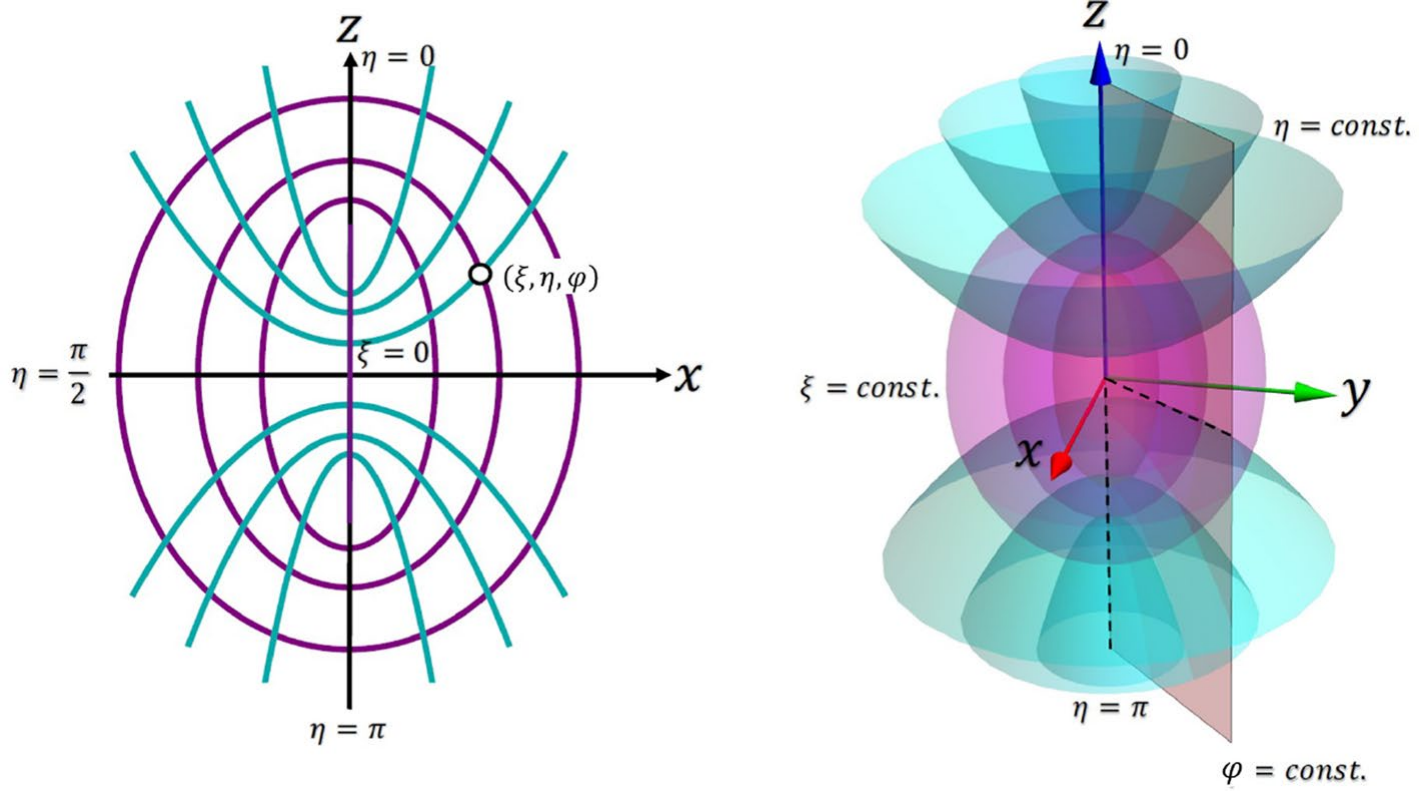
Multilayered confocal prolate spheroid in Cartesian coordinates. The spheroidal shape features two foci equidistant from the centre along the major axis. As the distance between these foci decreases, the spheroid becomes less elongated and forms a circle when the foci coincide at the centre. In confocal spheroids, all layers share the same foci

Figure 1 shows a prolate spheroid characterised by the coordinates ($\xi ,\eta ,\varphi$), where $\xi \in [0, \infty )$, $\eta \in [0,\pi ]$ and $\varphi \in [0,2\pi )$. The parameters of the spheroid are related to the Cartesian coordinates by:

1 $$\begin{aligned} \begin{aligned} x&= a\, {\text {sinh}}(\xi )\text {sin}(\eta )\text {cos}(\varphi )\\ y&= a\, {\text {sinh}}(\xi )\text {sin}(\eta )\text {sin}(\varphi )\\ z&= a\, \text {cosh}(\xi )\text {cos}(\eta ) \end{aligned} \end{aligned}$$

Where *a* is the focal length of the spheroid, given by:

2 $$\begin{aligned} \begin{aligned} a&= \sqrt{r_{\text {major}}^2-r_{\text {minor}}^2}\\ \end{aligned} \end{aligned}$$

$r_{\text {major}}$ ($r_{\text {minor}}$) is the length of the semi-major (minor) axis of the spheroid.

The surface of the spheroid is characterised by the parameter $\xi$, given by:

3 $$\begin{aligned} \begin{aligned} \xi = \text {cosh}^{-1}\left( \frac{r_{\text {major}}}{a}\right) \end{aligned} \end{aligned}$$

This work deals with multilayered *confocal* prolate spheroidal nanoparticles, which means different layers will have the same focal length *a* but different surface parameters $\xi$.

At first, we start our mathematical formulation from the simple case of a homogeneous spheroidal structure present in a parallel electric field $E_0$ incident along the $x-$axis and polarised along the major axis of the spheroid, the $z-$axis. The potential induced inside and outside of the spheroid can be obtained by solving Laplace’s equation:

4 $$\begin{aligned} \begin{aligned} \nabla ^2 \Phi =0 \end{aligned} \end{aligned}$$

The Laplacian is given by [13]:

5 $$\begin{aligned} \nabla ^2 \Phi = \frac{1}{h_1^2} \left( \frac{\partial ^2\Phi }{\partial \xi ^2}+\text {coth}(\xi )\frac{\partial \Phi }{\partial \xi }\right) +\frac{1}{h_2^2}\left( \frac{\partial ^2\Phi }{\partial \eta ^2} + \text {cot}(\eta )\frac{\partial \Phi }{\partial \eta }\right) + \frac{1}{h_3^2}\left( \frac{\partial ^2\Phi }{\partial \varphi ^2}\right) \end{aligned}$$

Where $h_1$, $h_2$, and $h_3$ are the scale factors that relate the basis vector of the spheroidal coordinates to the Cartesian coordinates system, calculated as follows:

6 $$\begin{aligned} \begin{aligned} h^2_1&= \left( \frac{\partial x}{\partial \xi }\right) ^2+ \left( \frac{\partial x}{\partial \eta }\right) ^2+\left( \frac{\partial x}{\partial \varphi }\right) ^2\\ h^2_2&= \left( \frac{\partial y}{\partial \xi }\right) ^2+ \left( \frac{\partial y}{\partial \eta }\right) ^2+\left( \frac{\partial y}{\partial \varphi }\right) ^2\\ h^2_3&= \left( \frac{\partial z}{\partial \xi }\right) ^2+ \left( \frac{\partial z}{\partial \eta }\right) ^2+\left( \frac{\partial z}{\partial \varphi }\right) ^2\\ \end{aligned} \end{aligned}$$

For the specific configuration considered in this work (a uniform incident electric field of amplitude $E_0$ polarised along the major axis of the prolate spheroid, i.e. along the Cartesian *z*-axis), the potential is independent of $\varphi$ and $h_1=h_2$ due to rotational symmetry. Hence the Laplace Eq. (4) can be simplified as:

7 $$\begin{aligned} \frac{\partial ^2\Phi }{\partial \xi ^2}+\text {coth}(\xi )\frac{\partial \Phi }{\partial \xi }+\frac{\partial ^2\Phi }{\partial \eta ^2} + \text {cot}(\eta )\frac{\partial \Phi }{\partial \eta }=0 \end{aligned}$$

The potential at any point is the sum of the potential of the incident field $\Phi _{in}$ and the perturbed potential induced by the spheroid $\Phi _P$. The potential of the incident field has the form:

8 $$\begin{aligned} \begin{aligned} \Phi _{in}&= - E_0z\\&=- E_0\,a \, \text {cosh}(\xi )\text {cos}(\eta ) \end{aligned} \end{aligned}$$

where *z* is substituted from Eq. (1).

The potential $\Phi _{in}$ can be rewritten in the following general form:

9 $$\begin{aligned} \begin{aligned} \Phi _{in}(\xi ,\eta )&= K_{in}F_1(\xi )G(\eta ) \end{aligned} \end{aligned}$$

Where

10 $$\begin{aligned} \begin{aligned} F_1(\xi )&=\text {cosh}(\xi )\\ G(\eta )&=\text {cos}(\eta )\\ K_{in}&=-aE_{0} \end{aligned} \end{aligned}$$

It is easy to verify that $\Phi _{in}$ is a solution to the Laplace’s Eq. (7).

To satisfy the boundary conditions, the perturbed potential $\Phi _p(\xi ,\eta )$ must have a similar form as $\Phi _{in}$, which we assume:

11 $$\begin{aligned} \Phi _p(\xi ,\eta ) = K_0F_2(\xi )G(\eta ) \end{aligned}$$

where $K_0$ is an amplitude coefficient. Substituting this expression into the Laplace’s Eq. (7), we get:

12 $$\begin{aligned} \frac{\partial ^2}{\partial \xi ^2}F_2(\xi )+\text {coth}(\xi )\frac{\partial }{\partial \xi }F_2(\xi )-2F_2(\xi )=0 \end{aligned}$$

Equation 12 is a second-order ordinary differential equation with two independent solutions. We already know one of these is $F_1(\xi )=\text {cosh}(\xi )$. The second solution can be obtained using the *reduction of order* method (see, e.g., page 122 in [14]). If $y_1$ is a solution of a second-order ordinary differential equation of the form:

13 $$\begin{aligned} \frac{\partial ^2y}{\partial x^2}+P(x)\frac{\partial y }{\partial x}+Q(x)y=0 \end{aligned}$$

A second independent solution $y_2$ can be obtained from:

14 $$\begin{aligned} y_2=y_1 \int \frac{e^{-\int P(x) dx}}{y_1^2}dx \end{aligned}$$

In our case, $x\equiv \xi$, $P(\xi )=\text {coth}(\xi )$, and $y_1(\xi )=F_1(\xi )=\text {cosh}(\xi )$. Therefore, the second solution is:

15 $$\begin{aligned} \begin{aligned} F_2(\xi )=\text {cosh}(\xi ) \int \frac{e^{-\int \text {coth}(\xi ) d\xi }}{\text {cosh}^2(\xi )}d\xi \end{aligned} \end{aligned}$$

which has an analytic solution of the form:

16 $$\begin{aligned} F_2(\xi )= F_1(\xi )\chi (\xi ) \end{aligned}$$

with the expression for $\chi (\xi )$ given by:

17 $$\begin{aligned} \chi (\xi )=- \left( \ln \left( \text {coth}\left( \frac{\xi }{2}\right) \right) -\text {sech}(\xi )\right) \end{aligned}$$

It is worth noting that $F_2(\xi )$ approaches zero at infinity but diverges at the center of the spheroid.

### Multilayered spheroid in a parallel field

In the above, we have derived the two independent analytical solutions of Laplace’s equation in the quasistatic approximation for a prolate spheroid, namely $F_1(\xi )G(\eta )$ and $F_2(\xi )G(\eta )$. The general solution of the potential of Laplace’s equation for a multilayered spheroid is a sum of the two solutions.

**Fig. 2:**
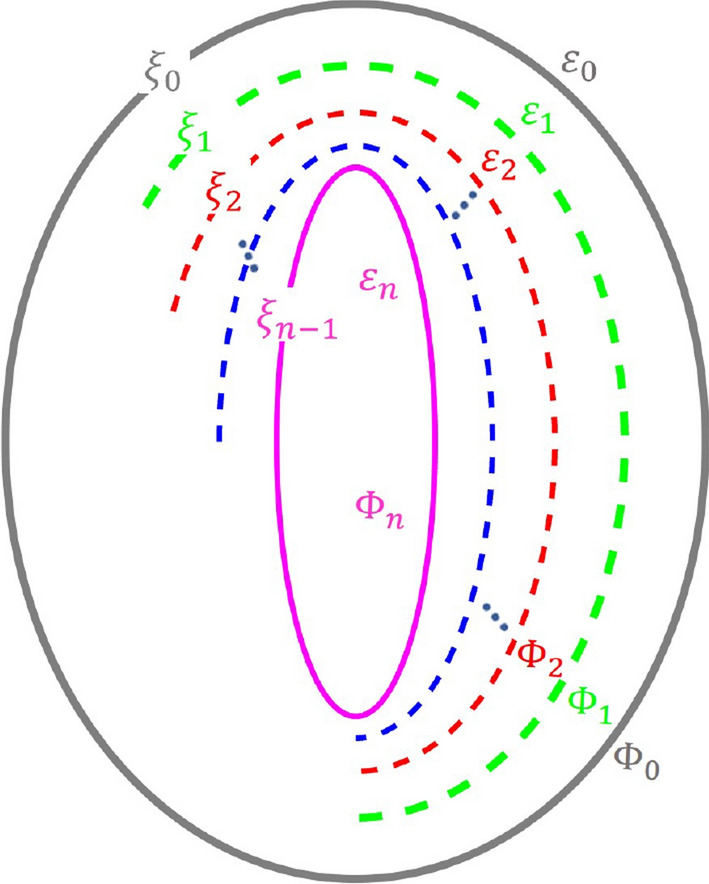
Multilayered confocal spheroids diagram showing the surfaces $\xi _i$, the optical constants $\varepsilon _i$, and the potential $\Phi _i$ in each layer

As shown in Fig. 2, the potential outside the spheroid is denoted as $\Phi _0$, and those in the layers l, 2, 3,... n, enclosed by the surfaces $\xi _0$, $\xi _1$, $\xi _2$,..., $\xi _{n-1}$, are denoted as $\Phi _1$, $\Phi _2$, $\Phi _3$,... and $\Phi _{n}$, respectively. The solutions of the potential in any region can be represented as a linear combination of $F_1(\xi )G(\eta )$ and $F_2(\xi )G(\eta )$:

18 $$\begin{aligned} \begin{aligned} \Phi _0&= K_{in}F_1(\xi )G(\eta )+K_0F_2(\xi )G(\eta )\\ \Phi _1&= K_1F_1(\xi )G(\eta )+K_2F_2(\xi )G(\eta )\\ \Phi _2&= K_3F_1(\xi )G(\eta )+K_4F_2(\xi )G(\eta )\\ \vdots \\ \Phi _{i}&= K_{2i-1}F_1(\xi )G(\eta )+K_{2i}F_2(\xi )G(\eta )\\ \vdots \\ \Phi _n&= K_{2n-1}F_1(\xi )G(\eta ) \end{aligned} \end{aligned}$$

where $K_i$ are the amplitude coefficients.

$\Phi _0$ is a superposition of the potential $\Phi _{in}$ of the incident field (Eq. 8) and the perturbed potential $\Phi _{p}$ of the spheroid (Eq. 11). As $F_2(\xi )$ is divergent at $\xi =0$ (the centre of spheroid), the potential in the core $\Phi _{n}$ only contains the term of $F_1(\xi )G(\eta )$ so that it is finite at the spheroidal centre.

For a $n-$ layer spheroid, there are 2*n* unknown coefficients $K_i$ ($i=0 \rightarrow 2n-1$). These coefficients can be determined from the boundary conditions at each surface. The boundary conditions at surface $\xi _i$ are:

19 $$\begin{aligned} \begin{aligned} \Phi _i|_{\xi \rightarrow \xi _i}&= \Phi _{i+1}|_{\xi \rightarrow \xi _i}\\ \varepsilon _i\frac{\partial }{\partial \xi }\Phi _i|_{\xi \rightarrow \xi _i}&= \varepsilon _{i+1}\frac{\partial }{\partial \xi }\Phi _{i+1}|_{\xi \rightarrow \xi _i}\\ \end{aligned} \end{aligned}$$

Equation 19 enforces (i) continuity of the electrostatic potential across each interface (no singular surface potential) and (ii) continuity of the normal component of the electric displacement field, i.e. $\varepsilon _i\,\partial \Phi _i/\partial n=\varepsilon _{i+1}\,\partial \Phi _{i+1}/\partial n$, assuming linear, isotropic media with no free surface charge at the interfaces. Since each interface is a surface of constant $\xi$, the outward normal is aligned with the $\xi$-direction and the normal derivative is proportional to $\partial /\partial \xi$, which leads to the second line of Eq. 19.

The boundary conditions result in two equations at each surface:

$$\underline{{\textbf {For the outermost surface }}(\xi _0)}$$

20 $$\begin{aligned} \begin{aligned} \chi (\xi _0)K_0 - K_1-\chi (\xi _0)K_2&= aE_0\\ \varepsilon _0A(\xi _0)K_0-\varepsilon _1K_1-\varepsilon _1A(\xi _0)K_2&=\varepsilon _0aE_0\\ \end{aligned} \end{aligned}$$

$$\underline{{\textbf {At any intermediate surface}} (\xi _i)}$$

21 $$\begin{aligned} \begin{aligned} K_{2i-1}+\chi (\xi _{ i})K_{2i} - K_{2i+1}-\chi (\xi _{i})K_{2i+2}&=0\\ \varepsilon _{i}K_{2i-1}+ \varepsilon _{i}A(\xi _{i})K_{2i}- \varepsilon _{i+1}K_{2i+1}-\varepsilon _{i+1}A(\xi _{i})K_{2i+2}&=0\\ \end{aligned} \end{aligned}$$

$$\underline{{\textbf {At the core surface }}(\xi _{n-1})}$$

22 $$\begin{aligned} \begin{aligned} K_{2n-3}+\chi (\xi _{n-1})K_{2n-2} - K_{2n-1}&=0\\ \varepsilon _{n-1}K_{2n-3}+ \varepsilon _{n-1}A(\xi _{n-1})K_{2n-2}- \varepsilon _{n}K_{2n-1}&=0\\ \end{aligned} \end{aligned}$$

Where:  $\displaystyle A(\xi _i)=\frac{1}{\text {sinh}^2(\xi _i)\text {cosh}(\xi _i)}+\chi (\xi _{i})$, resulting from the second boundary condition of Eq. (19).

The coefficients $K_i$ in the above equations can be determined by solving the linear equations obtained from the boundary conditions. Specifically, Eqs. 18–22 constitute a linear system of the form $M_2\vec {K}=M_1$, where $\vec {K}=[K_0, K_1, \dots , K_{2n-1}]^T$. $M_1$ and $M_2$ are matrices given by

$$ {\mathrm{M}}_{1} = \left( {\begin{array}{*{20}c} {aE_{0} } \\ {\varepsilon _{0} aE_{0} } \\ 0 \\ 0 \\ \vdots \\ 0 \\ 0 \\ \end{array} } \right), $$

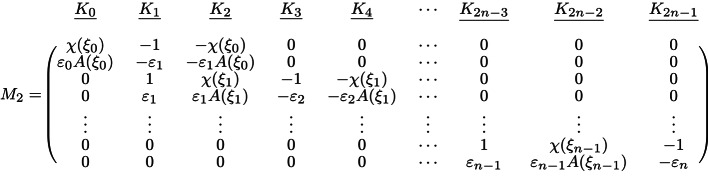


The coefficient $K_i$ can be determined from

23 $$\begin{aligned} \vec {K} = M_{2}^{-1}M_1 \end{aligned}$$

where $M_{2}^{-1}$ is the inverse matrix of $M_2$.

As will be shown in the following section, the main optical properties, such as scattering and absorption cross sections, are solely determined by the coefficient $K_0$, which can be conveniently calculated through the ratio of two matrix determinants.

24 $$\begin{aligned} K_0 = \frac{|M_{K_0}|}{|M_{2}|} \end{aligned}$$

$M_{K_0}$ is obtained by substituting the $K_0$ column in $M_2$ with $M_1$, which is given below
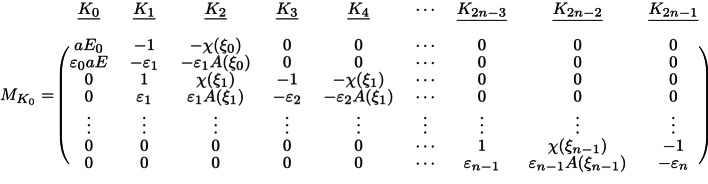


### Effective dipole moment

In the far-field region ($r>> r_{major}$), the effect of the spheroid is equivalent to an electric dipole with an effective dipole moment *P* located at the centre of the spheroid [15]. In the following, we will derive the formula for the effective dipole moment *P*.

Using the expressions of $F_2(\xi )$ (Eq. 16), $G(\eta )$ (Eq. 10) and *z* (Eq. 1), we can rewrite the perturbed potential (Eq. 11) as:

25 $$\begin{aligned} \begin{aligned} \Phi _p&= K_0F_2(\xi )G(\eta )\\&= K_0\text {cosh}(\xi )\text {cos}(\eta )\int \frac{1}{\text {cosh}^2(\xi )\text {sinh}(\xi )}d\xi \\&= \frac{K_0z}{a}\int \frac{1}{\text {cosh}^2(\xi )\text {sinh}(\xi )}d\xi \\ \end{aligned} \end{aligned}$$

In the far-field (large observer distance from the particle), $\xi \gg 1$, so we can make the following approximations:

26 $$\begin{aligned} \begin{aligned} \text {cosh}(\xi )&=\frac{e^\xi +e^{-\xi }}{2}\simeq \frac{e^\xi }{2}\\ \text {sinh}(\xi )&=\frac{e^\xi -e^{-\xi }}{2}\simeq \frac{e^\xi }{2}\\ \end{aligned} \end{aligned}$$

Therefore:

27 $$\begin{aligned} \begin{aligned} \Phi _{p}&\simeq \frac{K_0z}{a}\int \frac{1}{(\frac{1}{2}\,e^\xi )^3}d\xi \end{aligned} \end{aligned}$$

Performing the integration leads to:

28 $$\begin{aligned} \begin{aligned} \Phi _{p} \simeq -\frac{K_0z}{a}\frac{8}{3e^{3\xi }} \end{aligned} \end{aligned}$$

In the same far-field limit, the prolate spheroidal coordinate surfaces approach spheres, and the distance from the origin can be approximated by:

29 $$\begin{aligned} \begin{aligned} r&\simeq \frac{1}{2}ae^\xi \end{aligned} \end{aligned}$$

We can now rearrange the above result and substitute the parameters from Eq. 29 as follows:

30 $$\begin{aligned} \begin{aligned} \Phi _{p} \simeq -\frac{K_0z}{a}\left( \frac{a^3}{3(\frac{1}{2}ae^\xi )^3}\right) \simeq -\frac{K_0za^2}{3r^3} \end{aligned} \end{aligned}$$

The potential at a distance *r* due to an electric dipole located at the origin has the form [15]:

31 $$\begin{aligned} \begin{aligned} \Phi _{dipole} =\frac{\vec {P}\cdot \vec {r}}{4\pi \varepsilon _0r^3}=\frac{Pz}{4\pi \varepsilon _0r^3} \end{aligned} \end{aligned}$$

where $\vec {P}$ is the dipole moment. For the incident field polarised along the $z-$axis, the induced dipole moment will be along the $z-$axis, so $\vec {P} \cdot \vec {r}=Pz$ in Eq. (31). By comparing Eqs. (30 and 31), we reach the expression of the effective dipole moment produced by the multilayered spheroid as follows:

32 $$\begin{aligned} \begin{aligned} \frac{Pz}{4\pi \varepsilon _0r^3}&\simeq -\frac{K_0za^2}{3r^3}\\ \end{aligned} \end{aligned}$$

Thus:

33 $$\begin{aligned} \begin{aligned} P&= -\frac{4\pi }{3}a^2\varepsilon _0K_0 \end{aligned} \end{aligned}$$

### The effective polarisability

The polarisability $\alpha$ relates the induced dipole moment *P* to the incident field $E_0$ as:

34 $$\begin{aligned} P=\varepsilon _0{\alpha }E_0 \end{aligned}$$

Comparing this relation with Eq. (33), it follows straightforwardly that the effective polarisability can be expressed in the form:

35 $$\begin{aligned} \begin{aligned} \alpha = \frac{4\pi a^3}{3}\frac{K_0}{K_{in}} \end{aligned} \end{aligned}$$

In the above, we use the expression of $K_{in}$ shown in Eq. (10).

Having established the expression of effective polarisability, we can now characterise the scattering cross-section $\sigma _{sca}$ and absorption cross-section $\sigma _{abs}$ of multilayered confocal spheroids. For small nanoparticles, these are given by [15]:

36 $$\begin{aligned} \begin{aligned} \sigma _{sca}&= \frac{k^4}{6\pi }|\alpha |^2\\ \sigma _{abs}&= k\,Im|\alpha |\\ \end{aligned} \end{aligned}$$

Where $k=\frac{2\pi }{\lambda }$ is the wavevector of incident light; $|\alpha |$ refers to the magnitude of $\alpha$ and $Im|\alpha |$ refers to the imaginary part of $\alpha$.

Scattering and absorption are two very important optical properties of nanoparticles, which are central to many optical phenomena and applications and are often used to determine the plasmonic resonant wavelength of a nanoparticle. The above results show that, to characterise a multilayered confocal spheroid’s scattering and absorption properties, we only need to evaluate the coefficient $K_0$, which can be simply calculated according to Eq. (24).

## Results and discussion

The above analytical model is applicable to confocal spheroids of an arbitrary number of layers. Numerical evaluation of the derived closed-form expressions and generation of all spectra were performed in *Wolfram Mathematica* (v12.0) to investigate the optical properties of 2-, 3-, and 5-layer spherical and spheroidal systems (examples of a 4-layer spherical system are shown in Fig. S4), demonstrating the effectiveness and versatile capability of the simple analytic solutions. (No full-wave electromagnetic simulations such as FDTD/FEM are used in this work.) The spherical cases in the subsequent subsections are handled by making the spheroid’s minor and major axes almost identical.

### Comparison with the concentric ellipsoid analytical model

Prior to demonstrating the capabilities of the proposed analytical model, we first confirm its validity and accuracy by comparing the analytical expression of the polarisability derived in Eq. (35) with the polarisability for the case of core-shell concentric ellipsoidal geometry by Bohren & Huffman [15]:

37 $$\begin{aligned} \begin{aligned} \alpha = \frac{v((\varepsilon _1-\varepsilon _0)[\varepsilon _1+(\varepsilon _2-\varepsilon _1)(L^{(2)}_z-f L^{(1)}_z)]+f \varepsilon _1(\varepsilon _2-\varepsilon _1))}{([\varepsilon _1+(\varepsilon _2-\varepsilon _1)(L^{(2)}_z-f L^{(1)}_z)][\varepsilon _0+(\varepsilon _1-\varepsilon _0)L^{(1)}_z]+f L^{(1)}_z \varepsilon _1(\varepsilon _2-\varepsilon _1))} \end{aligned} \end{aligned}$$

Where $\varepsilon _i$ is the permitivity, the subscript $i=0$ represents the ambient medium, and $i=1,2$ represents the outer and inner ellipsoids, respectively. $v=4\pi a_1b_1c_1/3$ is the total volume of the particle, $f=a_2b_2c_2/a_1b_1c_1$ is the fraction of the volume occupied by the inner ellipsoid. *a* is the major axis, *b* and *c* are the minor axes. $L^{(1)}_z$ and $L^{(2)}_z$ are the geometrical factors along the major axis for the outer and inner ellipsoids, respectively:

38 $$\begin{aligned} \begin{aligned} L^{(i)}_z=\frac{a_ib_ic_i}{2}\int\limits _0^\infty \frac{1}{(a_i^2+q)f_i(q)}dq\,\,\,\,\,\,\,\,\,\,(i=1,2) \end{aligned} \end{aligned}$$

Where $$f_i(q)=\sqrt{(q+a_i^2)(q+b_i^2)(q+c_i^2)}$$.

We write the equivalent expression for the geometrical parameter $L^{(i)}_z$ in spheroidal coordinates in the form:

39 $$\begin{aligned} L^{(i)}_z=\text {sinh}^2(\xi _i)\text {cosh}(\xi _i)\int \frac{1}{\text {sinh}(\xi _i)\text {cosh}^2(\xi _i)}d\xi \end{aligned}$$

It is important to point out that the polarisability expression by Bohren & Huffman describes two *concentric* ellipsoids that form a uniformly coated particle. In contrast, the polarisability derived in this work describes *confocal* spheroids, which for the two-layer case results in a thicker coating layer along the minor axis for the spheroidal geometry $(b_i=c_i)$. Our model labels the major and minor axes as $a\,\equiv r_{\text {major}}$ and $b, c\,\equiv r_{\text {minor}}$, respectively.

**Fig. 3:**
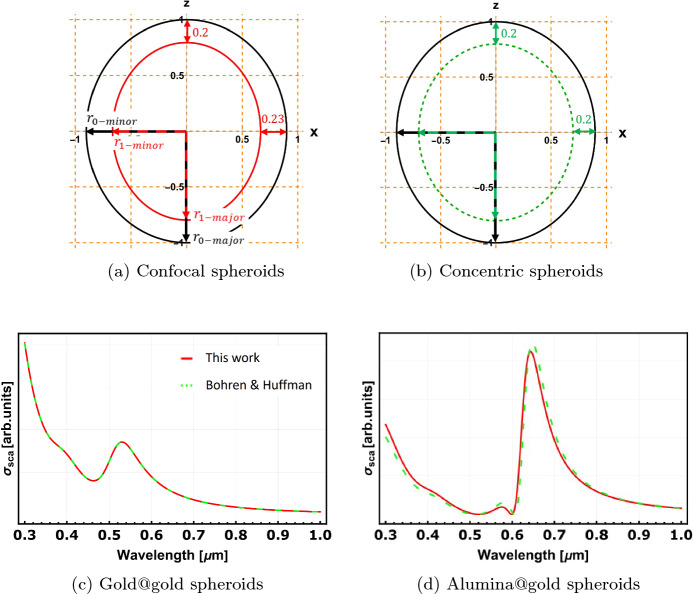
Comparison of analytical models. Diagram of a confocal spheroid (**a**) and a concentric spheroid (**b**). Scattering cross-sections of core-shell spheroidal nanoparticles where the two layers are made of the same materials (gold@gold) (**c**) and of different materials (alumina@gold) (**d**)

Figure 3a and b show the structures of a confocal and a concentric core-shell spheroidal nanoparticle, respectively. The major and minor axes of the outer spheroids for both structures are set to be equal $( r_{0-\text {major}}=1\, \mathrm{ \& } \, r_{0-\text {minor}}=0.9)$, where the units are arbitrary. The major axes for both structures’ inner spheroids are also equal $(r_{1-\text {major}}=0.8).$ As shown in Fig. 3a).

Because Bohren & Huffman [15] assumes *concentric* shells, while the present work treats *confocal* spheroids, a direct comparison for dissimilar core/shell materials would generally correspond to two *different physical structures* (slightly different layer thicknesses and aspect ratios). To isolate the analytical correctness of the polarisability expression, we therefore compare the two models in the homogeneous-limit by assigning the *same* material to both layers (e.g. Au@Au). In this case, both the confocal and concentric core-shell spheroids reduce to a homogeneous spheroid with identical outer geometry, and the scattering spectrum calculated using the polarisability defined in Eq. (35) (red solid line, Fig. 3c) is identical to that calculated using the Bohren & Huffman polarisability in Eq. (37) (green dashed line, Fig. 3c). We obtain the same agreement when both layers are assigned Al_2_O_3_ (not shown). This confirms the validity and accuracy of our analytical model in the limit where both formulations describe the same object. When the material of the inner spheroid is replaced with alumina (Al_2_O_3_) while keeping a gold outer layer, the results of the two models deviate slightly (Fig. 3d), which is expected because the confocal and concentric coated spheroids are no longer identical structures; for more eccentric geometries, the confocal–concentric mismatch in layer thickness/aspect ratio can increase and correspondingly larger deviations may occur. The optical constants for the materials used in all demonstrations are obtained from [16].

Overall, the above comparison provides a stringent analytical cross-check against established theory in the case where both models describe the same physical geometry (homogeneous-limit). As a further independent validation in a regime where a closed-form reference is available, we also verified the model in the case of a 3-layered spherical structure against the theoretical model of Sihvola and Lindell [12], finding indistinguishable results (Supplementary Information, Fig. S5) in the dipole limit. This further confirms the validity of our simple analytic solutions. The remaining demonstrations in Sects. 3.2−3.4 extend this validation by showing consistent scattering/absorption trends and stable numerical evaluation across multiple layer counts and parameter sweeps (2-, 3-, and 5-layer confocal systems), illustrating the generality of the closed-form formulation for multilayer confocal spheroids.

### 2-layer spherical nanostructure

**Fig. 4:**
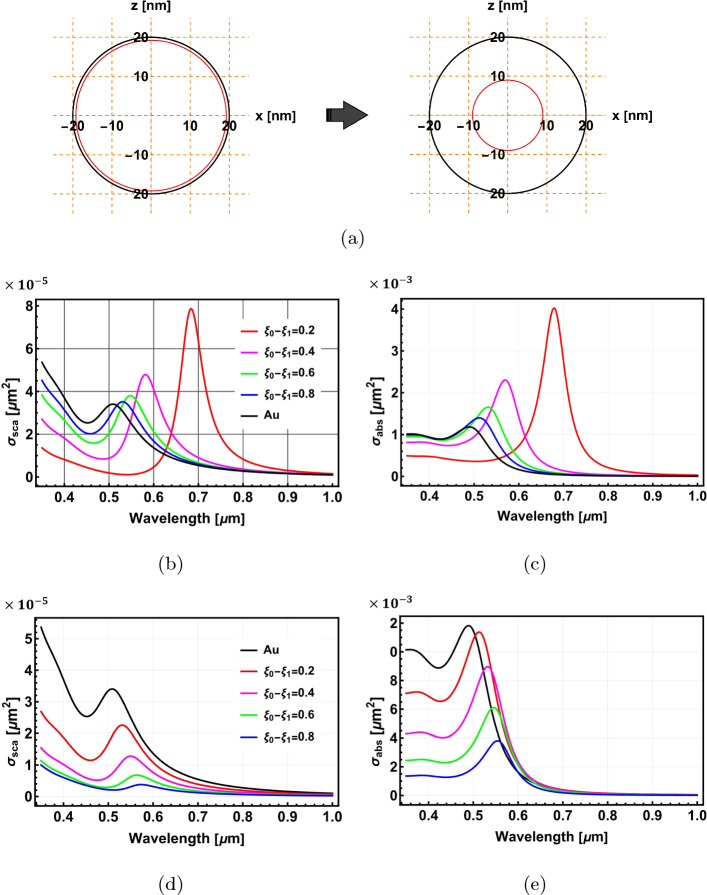
The optical properties of core@shell spherical nanoparticles. **a** Schematic of the geometry. **b** and **c** depict the scattering and absorption cross-section spectra of Al$_2$O$_3$@Au spherical nanoparticles with different shell thicknesses. **d** and **e** show the scattering and absorption cross-section spectra of Au@Al$_2$O$_3$ spheres, respectively

**Fig. 5:**
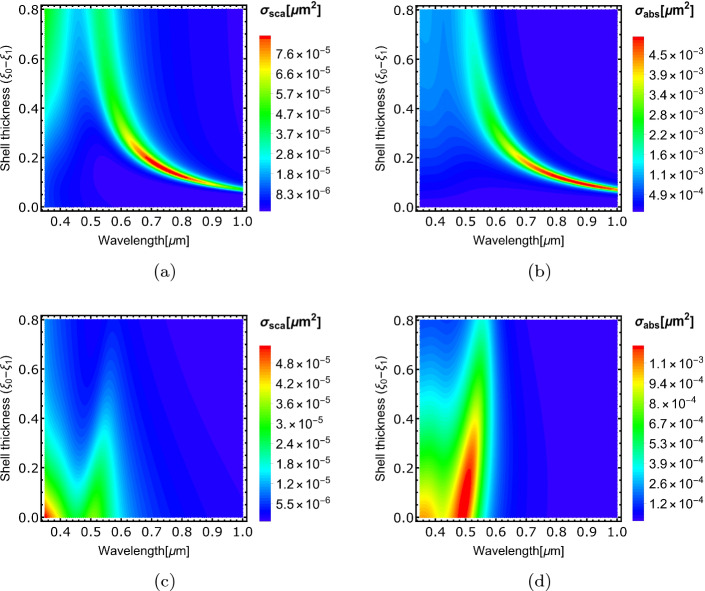
Density plots for the scattering and absorption cross-sections for Al$_2$O$_3$@Au (**a** and **b**) and Au@Al$_2$O$_3$ (**c** and **d**) core-shell spheres, respectively. The colour bars illustrate the significant variations in values when interchanging the materials of the core and shell. This demonstrates the sensitivity of metallic shell nanostructures to changes in their geometry

We begin by employing the model to compute the optical properties of conventional spherical core-shell nanostructures. Throughout this paper, we use the notion $M_n@...@M_1$ to denote a $n-$ layer structure, where $M_n$ represents the innermost core material and $M_1$ represents the outermost layer material. It is important to note that the $\text {cosh}(\xi _i)$ functions, which are used to define the surfaces of the spheroid in spheroidal coordinates, exhibit exponential growth. As a result, they are highly sensitive to changes in their arguments. Even small variations in the geometrical parameter $\xi _i$ can lead to substantial changes in the $\text {cosh}(\xi _i)$ value. This necessitates high precision and the use of more significant figures to accurately capture the shape of the ellipsoid. The structure of the spherical core-shell nanostructure is shown in Fig. 4a

Figure 4b and c present the calculated scattering and absorption cross-section spectra of Al$_2$O$_3$@Au spherical nanostructure, respectively, for different shell thicknesses. The spectra exhibit a pronounced resonant peak that blue-shifts and diminishes as the thickness of the gold shell increases and the size of the alumina core decreases. When the alumina core size is reduced to zero, the structure resembles a gold sphere, which shows a scattering resonant peak at $\lambda \simeq$ 0.52 $\mu$m (black curve, Fig. 4b). The calculations were repeated by interchanging the core and shell materials analysing Au@Al$_2$O$_3$ nanostructures (Fig. 4d and e). In this case, the resonant peak red-shifts and diminishes as the gold core size decreases and the alumina shell thickness increases. These characteristics can be better visualised in Fig. 5. Figure 5a and b and Fig. 5c and d are density plots of the scattering and absorption cross-sections for Al$_2$O$_3$@Au and Au@Al$_2$O$_3$ spherical nanostructures, respectively, which clearly show how the scattering and absorption cross-sections evolve with wavelength and shell thicknesses. The trends of plasmonic resonant peaks are clearly seen (See Fig. S1 in the Electronic Supplementary Material for further analysis for Al$_2$O$_3$@Ag and Ag@Al$_2$O$_3$ core-shell spherical nanostructures). It is noted that the optical properties of the core@shell spherical nanostructure with a metallic shell are more sensitive to changes in geometry, which will make it favourable in some applications, e.g., in localised surface plasmon resonance sensors.

###  2-layer spheroidal nanostructure

**Fig. 6:**
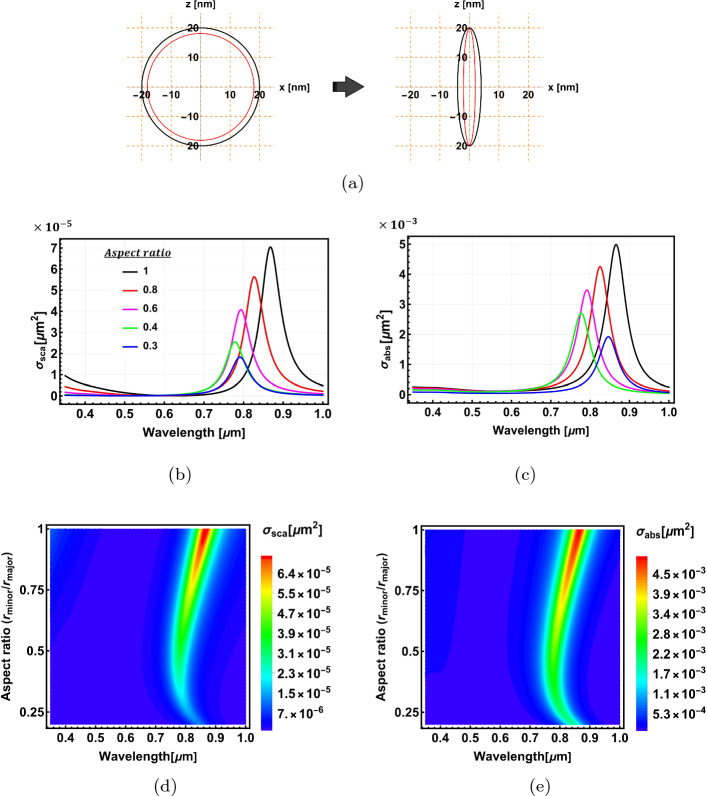
The optical properties of Al$_2$O$_3$@Au spheroidal nanoparticles. **a** shows the geometry of the nanospheroid. **b** and **c** depict the scattering and absorption cross-section spectra for different aspect ratios. **d** and **e** show density plots of the scattering and absorption cross-sections as a function of wavelength and aspect ratio, respectively. In figures (**b**–**e**), the resonant peak initially blue-shifts with decreasing aspect ratios due to the decrease in the volume size of the nanostructure. However, this trend reverses for smaller aspect ratios due to a competing effect from the geometrical parameter $L_z$ (see Eq. 39), which gets smaller with decreasing aspect ratios, red-shifting the resonant peaks to longer wavelengths

Here, we apply the analytical model to calculate the optical properties of a 2-layer spheroidal nanostructure. Figure 6a shows the structure’s geometry. The semi-major axis of the outer layer is fixed at $r_{0-\text {major}}=20$ nm. The surface parameter of the outer layer $\xi _0$ is varied from 5.1649 to 0.202733, while that of the inner layer is set to be $\xi _1=\xi _0 -0.1$. This changes the particle geometry from a near concentric sphere to an elongated confocal spheroid (Fig. 6a).

Figure 6b and c show, respectively, the scattering and absorption cross-sections for Al$_2$O$_3$@Au nanospheroids with different aspect ratios ($AR=r_{0-\text {minor}}/r_{0-\text {major}}$). An aspect ratio of 1 corresponds to the spherical nanostructure, illustrated with a black curve. The spherical shape has the highest magnitudes of scattering and absorption cross-sections, which decrease as the aspect ratio and, consequently, the geometrical cross-section decreases. The resonant peak initially blue-shifts with decreasing aspect ratios due to the decrease in the volume size of the nanostructure. However, this trend reverses for smaller aspect ratios due to a competing effect from the geometrical parameter $L_z$ (see Eq. 39), which gets smaller with decreasing aspect ratios, red-shifting the resonant peaks to longer wavelengths. The general trends of the optical properties are depicted in Fig. 6d and e.

**Fig. 7:**
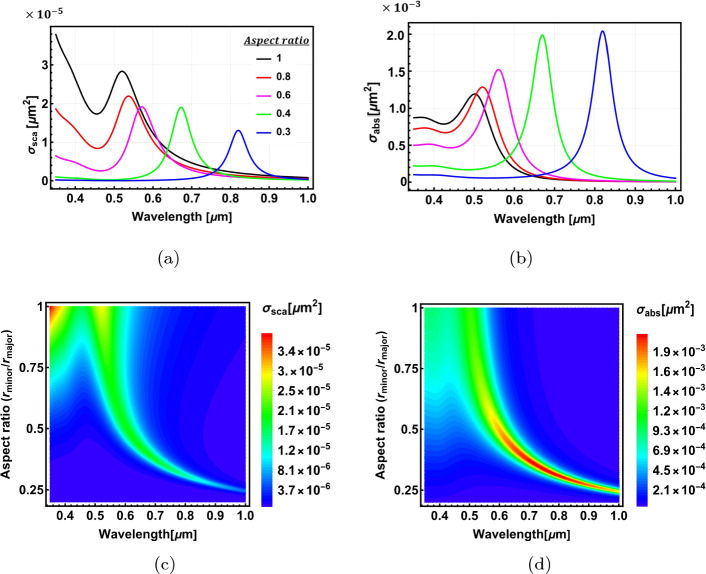
The optical properties of Au@Al$_2$O$_3$ core@shell spheroidal nanoparticles. **a** and **b** show the scattering and absorption cross-sections as a function of wavelength for different aspect ratios, respectively. **c** and **d** show density plots of the scattering and absorption cross-sections as a function of wavelength and aspect ratio, respectively

As the previous section shows, swapping the core and shell materials will generally lead to different optical properties. In Fig. 7a–d, we show the optical properties of Au@Al$_2$O$_3$ nanospheroids. The resonant peaks red-shift as the aspect ratio decreases, which, however, does not show the reversal behaviour observed in the Al$_2$O$_3$@Au nanospheroids (Fig. 6d and e). The resonant peaks are also more sensitive to the change of aspect ratio than the Al$_2$O$_3$@Au nanospheroids. This is because, in the case of the Au core, the effect of the geometrical parameter $L_z$ is aligned with the general trend of resonance shift with decreasing aspect ratio. Hence the overall effect is enhanced and becomes more pronounced. (See Fig. S2 in the Electronic Supplementary Material for further analysis for Al$_2$O$_3$@Ag and Ag@Al$_2$O$_3$ core-shell spheroidal nanostructures)

### 3-layer spherical nanostructure

Multilayered structures with more than two layers offer more freedom to tune the optical properties of nanoparticles. Furthermore, they could provide additional control of the functionality of the nanostructure by coating the metallic shell with a layer of material that can enhance specific properties (e.g. optical, thermal, physical, chemical or mechanical) or interact with the surrounding medium in a certain way [17]. In this section, we demonstrate the capability of the proposed analytical model for analysing the optical properties of a 3-layer Au@Al$_2$O$_3$@Au spherical system. The surface parameters $\xi _0=4.1469$ and $\xi _2=3.4$ of the outermost and the innermost layers are kept fixed, while the surface parameter $\xi _1$ of the intermediate layer is varied from 4.0969 to 3.5469. The radii of the outermost and innermost layers are fixed at 20 nm and 9 nm, respectively. The radius of the intermediate layer varies from 19 to 11 nm.

**Fig. 8:**
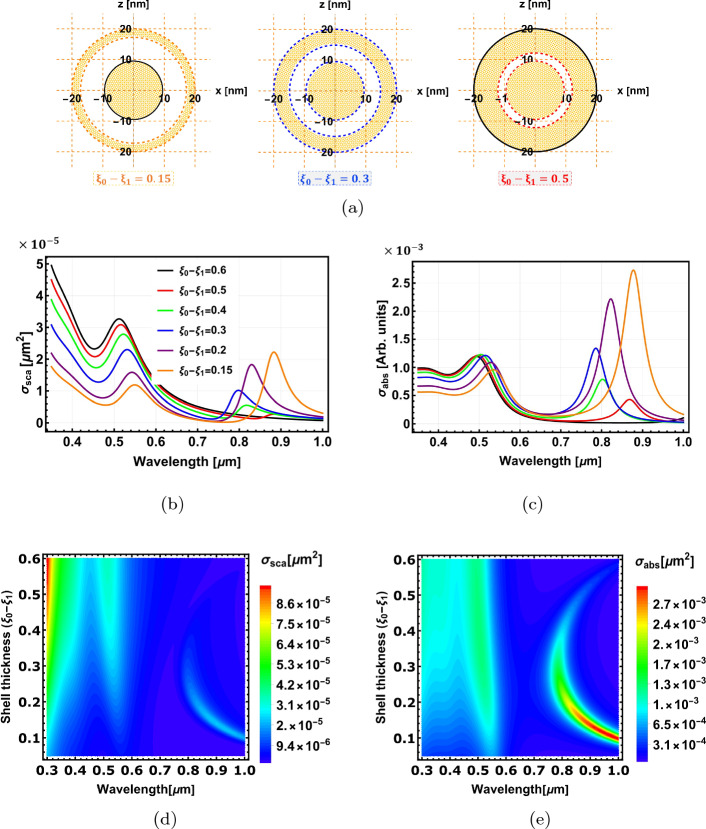
The optical properties of Au@Al$_2$O$_3$@Au spherical nanostructure. **a** the geometrical configurations of three different layer thicknesses. **b** and **c** are the scattering and absorption cross-section spectra for different shell thicknesses. **d** and **e** are plot maps of the scattering and absorption cross-sections, respectively

Figure 8a shows schematics of three different configurations represented by the surface parameter difference $\xi _0-\xi _1$. Figure 8b and c show the scattering and absorption cross-section spectra for different shell thicknesses, respectively. Figure 8d and e are the plot maps for those cross-sections as a function of wavelength and shell thickness.

The scattering and absorption cross-section spectra are characterised by two strong peaks in the visible and near-infrared spectral regions. The separation between the two peaks widens as the distance between the metallic surfaces decreases as a result of the interaction (hybridisation) between the plasmons of the metallic surfaces [18–20]. An interesting point to note is that the long wavelength peak blue-shifts and diminishes as the shell thickness ($\xi _0-\xi _1$) increases from 0.15 up to 0.3. However, the trend reverses beyond this turning point, and the peak starts to red-shift with a further increase in shell thickness. This is clearly witnessed in Fig. 8d and e.

This reverse trend occurs as initially (for $\xi _0-\xi _1$ = 0.15), the metal shell thickness is small, and the outer surfaces $\xi _0$ and $\xi _1$ are close to each other (first configuration in Fig. 8a), which results in strong interaction between these two surfaces, and hence, strong peak and large separation. As the metal shell thickness increases and the spacer size decreases, the interaction of the electrons between the surfaces $\xi _0$ and $\xi _1$ weakens; hence, the peaks’ separation diminishes and the longer wavelength peak blue-shifts. When the shell thickness reaches $\xi _0-\xi _1$ = 0.3, the distances between the three metallic surfaces are almost equal (second configuration in Fig. 8a). This is a turning point, as for shell thickness $\xi _0-\xi _1>$ 0.3, the surfaces $\xi _1$ and $\xi _2$ get closer to each other, and the plasmon interaction occurs between these two surfaces. The peak separation increases, and the longer wavelength peak starts to red-shift again, but with smaller magnitudes due to the smaller sizes of the interacting surfaces and the screening effect of the outer metal. (See Fig. S3 in the Electronic Supplementary Material for further analysis of 3-layer spherical nanostructures with Ag, Al, and Cu metals and Al$_2$O$_3$ spacer layer)

###  5-layer spheroidal nanostructure

**Fig. 9:**
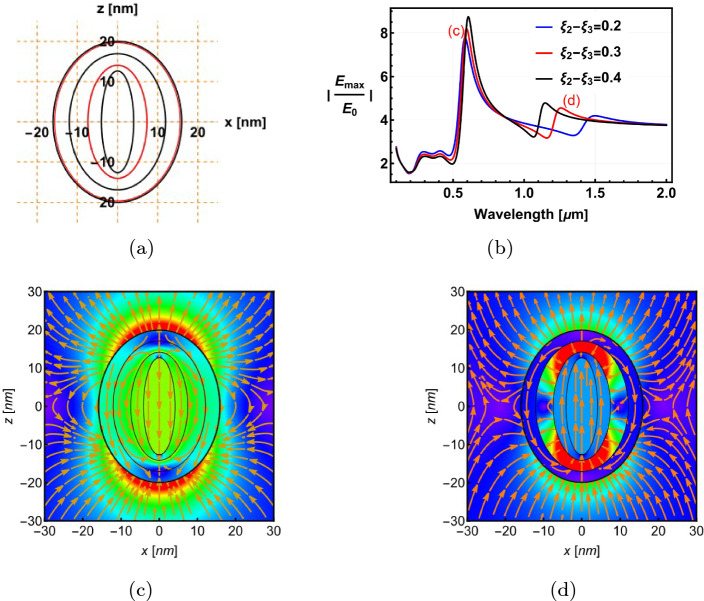
Hybrid 5-layer core@shell spheroidal nanoparticle designed for tuning and controlling the localised surface plasmonic resonance and the spatial distribution of the electric field. **a** shows the geometry and materials of the nanoparticle. The incident electric field (E$_0$) is polarised along the z-axis. **b** is a plot of the field enhancement (E$_{max}$/E$_0$), showing four distinct peaks resulting from the hybridisation [18–20] of the different layers. **c** and **d** show the electric field distribution plotted for the peaks at  600 nm and  1300 nm, respectively. The electric field vectors are shown in orange arrows originating from positive charges and ending on negative charges, which indicates the charge distribution in the multilayered nanoparticle

The final study shows the capability of our analytical model to analyse more complicated structures with a large number of layers and to evaluate the properties of electric fields (which can be calculated straightforwardly from the potential according to $E=-\nabla \Phi)$. As an example, we investigate a 5-layer Al$_2$O$_3$@Au@Al$_2$O$_3$@Au@Al$_2$O$_3$ spheroidal nanostructure (Fig. 9a). The surface parameters for three specific configurations are shown in Table 1. Only the parameter $\xi _3$ is varied to tune the thickness of the middle Al$_2$O$_3$ spacer layer.

**Table 1:** The surface parameters of the multilayered spheroid in Fig. 9a from the outer layer to the inner layer

Graph	$\xi _0$	$\xi _1$	$\xi _2$	$\xi _3$	$\xi _4$
Black	1.09861	1.08861	0.888612	0.688,612	0.338612
Red	1.09861	1.08861	0.888612	0.588,612	0.338612
Blue	1.09861	1.08861	0.888612	0.488,612	0.338612

An incident electromagnetic wave with a magnitude of E$_0$ = 1 V/m propagates in the $x-$direction and polarised along the major axis ($z-$axis) of the multilayered nanospheroid. Figure 9b plots the maximum field enhancement as a function of the wavelength of incident light for the three configurations presented in Table 1. Four resonant peaks are clearly visible (two small peaks between 0.2 and 0.5 $\mu$m and two pronounced peaks between 0.5 $\mu$m - 1.6 $\mu$m, respectively) as a result of the hybridisation between the plasmonic modes of two Au shells [18–20]. In analogue to the analysis of the 3-layer system, when the Al$_2$O$_3$ spacer layer $\xi _2-\xi _3$ decreases, the metallic surfaces $\xi _2$ and $\xi _3$ get closer to each other, and the interaction (hybridisation) of their free electrons becomes stronger. This widens the separation between the two strong resonant peaks. The magnitude of the long wavelength peak is diminished due to the screening effect by the outer metal layer.

Moreover, we plot the electric field distribution in the nanostructure described by the red curve (second configuration in Table 1) at the resonant wavelengths around 0.6 $\mu$m and 1.3 $\mu$m in Fig. 9c and d), and the polarisation of the near fields is parallel to the polarisation of the incident wave. This mode represents the higher energy (bonding) mode as described in the hybridisation model in the literature [18–20]. On the other hand, for the resonance at the long wavelength of 1.3 $\mu$m, the electric field enhancement mostly occurs in the Al$_2$O$_3$ spacer region $\xi _3$ (Fig. 9d). This mode represents the lower energy (anti-bonding) mode. Here, we show that with multilayered structures, more complex resonance spectra containing multiple resonant frequencies can be achieved, and the optical energy can be tuned to be selectively concentrated in specified regions within the nanostructure dependent on the resonant frequency.

Applicability and limitations. The present formulation is derived within the quasi-static approximation, i.e. for particle dimensions that are small compared with the wavelength such that retardation effects can be neglected. The analytical development assumes linear, isotropic constituent media described by local (frequency-dependent) relative permittivities $\varepsilon _i(\omega )$, and it treats a uniform incident field polarised along the spheroid major axis (Cartesian $z$-axis), which yields an axisymmetric potential $\Phi$ that is independent of the azimuthal angle $\varphi.$ Other excitation orientations can be addressed by decomposing the incident field into components along the principal axes and superposing the corresponding responses. In principle, the closed-form expressions apply to an arbitrary number of confocal layers; however, for very large layer counts or extreme permittivity contrasts (e.g. very thin layers and/or strongly dispersive metals), the associated linear system and determinant evaluations can become numerically ill-conditioned. In such cases, convergence should be verified (and higher-precision arithmetic can be used) when computing spectra and near-field quantities. In practical terms, this regime and formulation are particularly useful for rapid parameter sweeps and inverse-design-style screening of multilayer spheroidal nanostructures (materials, shell thicknesses, and aspect ratio) to target specific response metrics such as scattering, absorption, and near-field enhancement. Because the expressions reduce evaluation to compact matrix operations, they can be implemented in lightweight environments (e.g. Mathematica) and can be considerably faster than repeated full-wave simulations when exploring large design spaces or very thin-layer configurations.

##  Conclusion

In summary, we provide simple analytical solutions to Laplace’s equation for multilayered confocal prolate spheroids in the quasi-static approximation for incident light polarised along the spheroid’s major axis. The resulting field potential is a sum of two analytic functions, with amplitude coefficients derived from basic matrix formulations that fully define the spheroid’s optical properties. Notably, the overall optical properties, such as scattering and absorption, are solely dependent on a single amplitude coefficient, $K_0,$ calculated as the ratio of two matrix determinants. This simple analytical model significantly simplifies the complexity of scattering problems associated with multilayered spheroidal nanoparticles, which are typically challenging to address, enabling rapid assessment of their optical properties. The model offers a facile and powerful tool for customising complex optical properties for a wide array of applications. Numerical results for 2-, 3-, and 5-layer spherical and spheroidal nanostructures are provided to demonstrate the effectiveness of our proposed analytical model. Although our model focuses on prolate spheroids, extending it to oblate spheroids is straightforward and will be addressed in future publications.

## Supplementary Information

Supplementary file1.

## Data Availability

All data generated or analysed during this study are included in this published article and its supplementary information files. To request data supporting the findings, inquire with M.A. andF.H.

## Code availability

To request codes for data processing inquire with M.A.
