# Supplementary material for: Simple analytical solutions for the optical properties of multilayered confocal prolate spheroids in the quasistatic approximation
Source: Discov Nano. 2026 May 27;21(1):221. doi: 10.1186/s11671-026-04607-5 (PMC13216373; doi:10.1186/s11671-026-04607-5)
Supplement: Supplementary file 1 — Supplementary file1. [file 11671_2026_4607_MOESM1_ESM.pdf]

Supplementary Information

Simple Analytical Solutions for the Optical Properties of Multilayered Confocal Prolate Spheroids in the Quasistatic Approximation

Mugahid Ali[1,2*] and Fumin Huang[3†]

[1*]Centre for Doctoral Training in Integrated Photonics and Advanced Data Storage (PIADS), School of Mathematics and Physics, Queen's University Belfast, Belfast, BT7 1NN, United Kingdom.
[2]Currently with the School of Physics and CRANN, Trinity College Dublin, Dublin2, D02 PN40, Ireland.
[3]Centre for Quantum Materials and Technologies, School of Mathematics and Physics, Queen's University Belfast, Belfast, BT7 1NN, United Kingdom.

*Corresponding author(s). E-mail(s): alim2@tcd.ie;
Contributing authors: f.huang@qub.ac.uk;
†These authors contributed equally to this work.

This Electronic Supplementary Material presents additional figures that highlight the application of our analytical model in calculating the optical properties of multi-layered nanostructures. While the primary manuscript primarily discussed the use of gold (Au) as the material of the core or the shell of the nanostructure to showcase the capabilities of the model, this document broadens the scope to include other plasmonic materials, namely silver (Ag), aluminium (Al), and copper (Cu). The optical constants for these materials were sourced from [1].

The inclusion of these diverse materials provides a more comprehensive validation of our model, highlighting its versatility and robustness. The model's ability to rapidly produce results for nanostructures with varying numbers of layers and different materials demonstrates its potential as a powerful tool in the fields of plasmonics and nanophotonics. This capability facilitates the thorough exploration and nuanced analysis of a vast array of multilayer configurations, making it an invaluable asset for researchers aiming to optimise optical properties for a variety of applications from medical imaging to photovoltaic devices.

By broadening the analysis to encompass a diverse array of materials, this Electronic Supplementary Material not only augments the primary manuscript but also deepens insights into the impact of different materials on the optical properties of multilayer nanostructures across various geometrical configurations. This enhanced understanding facilitates a more comprehensive exploration of how structural variations can tailor optical responses in complex systems.

S1 Analysis of Optical Properties of 2-layer Spherical Nanostructure

The structure of the spherical core-shell nanostructure is illustrated in Figure S1a. For this study, the surface parameter $\xi_0 = 8.75219$ and the semi-major axis $a_{0-major} = 20$ nm of the outer shell remain constant, while the inner core surface parameter, ξ_1, is varied from 8.75219 to 7.75219. This adjustment changes the radius of the inner core from 20 nm to 9 nm, effectively altering the core-shell ratio and influencing the optical behaviour of the nanostructure. For further details on converting between Spheroidal and Cartesian coordinates, please refer to Equation 3 in the primary manuscript.

Figures S1b and S1c display density plots of the scattering cross-section spectra (σ_{sca}) and absorption cross-section spectra (σ_{abs}) for Al_2O_3@Ag spherical nanostructures over varying shell thicknesses. The plots reveal a pronounced resonant peak in the scattering spectra that shifts toward shorter wavelengths (blue-shifts) as the thickness of the silver shell increases and the size of the alumina core decreases. In particular, when the alumina core is completely absent, the nanostructure behaves as a pure silver sphere, showing scattering and absorption resonant peaks at approximately $\lambda \simeq 0.365$ μm and $\lambda \simeq 0.360$ μm respectively. The intensity of these peaks, which are mapped on the colour scale to the right of the figures, diminishes towards the near-infrared spectrum.

It should be noted that the scattering cross-section peak magnitude of Al_2O_3@Ag is larger than that of Al_2O_3@Au by an order of magnitude. This significant increase highlights the enhanced plasmonic properties of silver compared to those of gold. In

contrast, the absorption cross section shows a minor increase, approximately doubling (see Figure 5 in the primary manuscript for comparison).

The calculations were repeated by interchanging the core and shell materials, analysing Ag@Al_2O_3 nanostructures (Figures S1d and S1e). In this case, the resonant peak red-shifts and diminishes as the silver core size decreases and the alumina shell thickness increases. The red-shift is a result of the reduced effective plasmonic volume as the core size decreases. This shift is significantly less sensitive to variations in shell thickness and core size in this configuration, indicating different plasmonic coupling dynamics compared to the Al_2O_3@Ag configuration.

These figures clearly demonstrate how the scattering and absorption cross-sections evolve with wavelength and shell thickness for spherical core-shell nanostructures. This highlights the model's efficiency in handling materials with different dielectric functions, analysing their performance, and providing a powerful tool for investigating and designing devices for various plasmonic applications. By showcasing the versatility of the model in dealing with different material combinations, we underscore its potential as an invaluable resource in the field of nanophotonics, facilitating the development of advanced nanostructures with tailored optical properties.

S2 Analysis of Optical Properties of 2-layer Spheroidal Nanostructure

In this section of the supplementary document, we delve into the optical characteristics of Al_2O_3@Ag core-shell spheroidal nanostructures, examining how these properties vary with wavelength and aspect ratio. This analysis provides deeper insights into the behaviour of plasmonic materials under different geometric constraints and offers guidance for optimising nanostructure design for specific applications.

Figure S2 illustrates the impact of wavelength and aspect ratio on the optical properties of these nanostructures, providing a detailed visual representation of their optical behaviour under varying conditions.

Figure S2a illustrates a 2-layer spheroidal nanostructure where the outer layer maintains a constant semi-major axis ($r_{0-major}$ = 20 nm). The surface parameter of the outer layer, ξ_0, ranges from 5.1649 to 0.202733, and the inner layer's surface parameter, ξ_1, is consistently set at $\xi_0 - 0.1$. This parameter adjustment gradually transforms the nanostructure from a nearly concentric sphere to an elongated confocal spheroid, providing a visual demonstration of the geometric flexibility inherent to this model.

Figures S2b and S2c detail the scattering (σ_{sca}) and absorption (σ_{abs}) cross-sections for Al_2O_3@Ag nanospheroids across various aspect ratios (AR = $r_{0-minor}/r_{0-major}$), where an AR of 1 indicates a perfect spherical form. These figures reveal that the maximum scattering and absorption cross-sections are observed when the nanostructure is spherical, with values diminishing as the aspect ratio decreases. This reduction in cross-sections correlates with a decrease in the geometric cross-section, illustrating the impact of shape on the optical responses of the materials.

For Al_2O_3@Ag nanospheroids, an initial blue-shift in the resonant peak is observed as the aspect ratio decreases, which is attributed to the reduced volume of the nanostructure. However, as the aspect ratio becomes smaller than approximately 0.3, this trend reverses due to the reduction in the geometrical parameter L_z (refer to Equation 41 in the primary manuscript), resulting in a red shift of the resonant peaks towards longer wavelengths.

Figures S2d and S2e focus on the optical properties of Ag@Al_2O_3 nanospheroids. Unlike the Al_2O_3@Ag configurations, the resonant peaks in these figures consistently red-shift with a decreasing aspect ratio without the observed reversal. This distinct behaviour in Ag@Al_2O_3 nanostructures highlights how the geometrical parameter L_z aligns with the general resonance shift trend, resulting in a more pronounced and sensitive response to changes in aspect ratio.

Furthermore, a comparative analysis of spheroidal core-shell configurations using Ag and Au plasmonic materials reveals that Ag results in a significantly higher scattering cross-section compared to Au, with only a minor change in the absorption cross-section. This comparison underlines the superior scattering capabilities of Ag in these configurations and points to its potential for enhanced performance in specific plasmonic applications. Additionally, the use of plasmonic material as the core increases the tunability of the nanostructure, enhancing the capacity to tailor optical properties to meet specific needs in plasmonic device design.

This section underscores the advanced capabilities of our analytical model in exploring and exploiting the intricate relationship between nanostructure geometry, material composition, and optical properties, thereby facilitating the design of optimised devices for a range of plasmonic applications.

S3 Analysis of Optical Properties of 3-layer Spherical Nanostructure

Multilayered structures, consisting of more than two layers, provide substantial flexibility in modulating the optical properties of nanoparticles. These structures also enable enhanced control over nanostructure functionality by incorporating a thin material layer over the metallic shell, which can significantly improve specific properties such as optical, thermal, physical, chemical, or mechanical attributes or interact distinctively with the surrounding environment.

In this supplementary section, we delve deeper into expanding the proposed analytical model to study the optical properties of three-layer spherical systems using silver (Ag), aluminium (Al), and copper (Cu) as the plasmonic materials, denoted as Ag@Al_2O_3@Ag, Al@Al_2O_3@Al, and Cu@Al_2O_3@Cu. We maintain fixed surface parameters, $\xi_0 = 4.1469$ and $\xi_2 = 3.4$, for the outermost and innermost layers, respectively. In contrast, the surface parameter ξ_1 of the intermediate layer (spacer layer) varies from 4.0969 to 3.5469. These settings correspond to the radii of the outermost and innermost layers being fixed at 20 nm and 9 nm, respectively, with the radius of the intermediate layer varying from 19 nm to 11 nm.

Figure S3a illustrates schematics of three different configurations distinguished by the surface parameter difference $\xi_0 - \xi_1$. Plot maps showcasing the scattering

(σ_{sca}) and absorption (σ_{abs}) cross-section spectra as a function of wavelength and shell thickness are displayed in Figures S3b and S3c, S3d and S3e, and S3f and S3g for Ag@Al_2O_3@Ag, Al@Al_2O_3@Al, and Cu@Al_2O_3@Cu multilayered spherical nanostructures, respectively.

The optical behaviour of these systems typically features two pronounced peaks in the visible (bonding mode) and near-infrared (anti-bonding mode) regions. The separation between these peaks increases as the distance between the metallic surfaces decreases, driven by plasmonic interaction at the intermediate layer (hybridisation) [2–4]. Notably, the long-wavelength peak (anti-bonding mode) initially blue-shifts and then diminishes as the shell thickness ($\xi_0 - \xi_1$) increases from 0.08 to 0.3. Beyond this critical point, the peak undergoes a red shift with further increases in shell thickness, as observed across all anti-bonding modes in the hybridisation splittings depicted in Figure S3.

This reverse trend occurs only for the anti-bonding mode, as initially (for $\xi_0 - \xi_1 = 0.15$), the metal shell thickness is small, and the outer surfaces ξ_0 and ξ_1 are close to each other (the orange-dashed lines in the first configuration in Figure S3a), which results in strong interaction between these two surfaces and hence, strong peak and large separation. As the metal shell thickness increases and the spacer size decreases, the interaction of the electrons between the surfaces ξ_0 and ξ_1 weakens; hence, the peaks' separation diminishes and the longer wavelength peak blue-shifts. When the shell thickness reaches $\xi_0 - \xi_1 = 0.3$, the distances between the three metallic surfaces are almost equal (the blue-dashed lines in the second configuration in Figure S3a). This is a turning point, as for shell thickness $\xi_0 - \xi_1 > 0.3$, the surfaces ξ_1 and ξ_2 get closer to each other (the red-dashed lines in the third configuration in Figure S3a), and the plasmon interaction occurs between these two surfaces. The peak separation increases, and the longer wavelength peak starts to red-shift again, but with smaller magnitudes due to the smaller sizes of the interacting surfaces and the screening effect of the outer metal.

Comparative analysis of the optical properties of Ag, Al, and Cu in a three-layer spherical configuration reveals that systems with Ag demonstrate the highest scattering and absorption cross-section magnitudes. The optical performance of Cu, noted for a weaker peak splitting but a narrower and slightly stronger resonance in the infrared region, marginally surpasses that of Au (see Figures 8(d) and 8(e) in the primary manuscript for this comparison). Meanwhile, the higher energy peak (bonding mode) is consistently blue-shifted and enhanced as the spacer layer thickness increases; nevertheless, this shift is less sensitive to metallic surface separation. For Al@Al_2O_3@Al it falls outside the studied wavelength range ($\lambda < 0.3\ \mu m$), with the maximum scattering and absorption cross-sections occurring when the shell thickness is between $0.1 < \xi_0 - \xi_1 < 0.3$ (Figures S3d and S3e).

The consistent trend in peak shifts and intensity changes due to variations in shell thickness across these materials underscores the robustness, versatility, and practicality of our analytical model in evaluating a diverse array of materials and geometrical parameters, enhancing its utility in the design and optimisation of plasmonic nanostructures.

S4 Additional validation: 4-layer confocal spheroid

To address reviewer requests for numerical validation beyond the two-, three-, and five-layer cases presented in the main manuscript, we include here a representative four-layer Au@Al_2O_3@Au@Al_2O_3 spherical structure. For consistency with the notation used in the manuscript and in the axis labels of Fig. S4, we define four confocal interfaces $\xi_0, \xi_1, \xi_2, \xi_3$, where ξ_0 is the outermost surface and ξ_3 is the innermost interface. The first three interfaces are kept constant at $\xi_0 = 7.25433$, $\xi_1 = 7.20433$, and $\xi_2 = 7.15433$, while the innermost interface is swept from $\xi_3 = 7.10433$ to $\xi_3 = 6.55433$. This corresponds to varying the effective inner-shell thickness parameter $(\xi_2 - \xi_3)$ from 0.05 to 0.60 (as shown on the vertical axis), while maintaining an almost circular cross-section in Cartesian coordinates. Figure S4(a,b) show the initial and final configurations and Figure S4(c,d) are density plots of the scattering and absorption cross-section spectra, respectively, for Au@Al_2O_3@Au@Al_2O_3 nanostructure. The absorption map shows stronger response for thinner effective shells (smaller $\xi_2 - \xi_3$), corresponding to a large Au core, while the scattering map exhibits an optimised band at intermediate thickness values (roughly $\xi_2 - \xi_3 \sim 0.1$–0.4) depending on wavelength.

S5 Additional validation: layered-sphere limit (comparison with Sihvola and Lindell)

To provide an additional independent cross-check in a case where existing closed-form results are available, we validated the spherical (nearly concentric) limit of our formulation against Eq. (2) in Sihvola and Lindell [5], which gives an explicit dipole polarisability for layered ellipsoids (including the multilayered spherical limit). For reference, we reproduce below the nested form of Eq. (2) as presented in [5] for a multilayered sphere (outer radius a_1 and successive interface radii $a_2, a_3, \ldots$, permittivities $\varepsilon_0, \varepsilon_1, \varepsilon_2, \ldots$):

$$\alpha = 4\pi\varepsilon_0 \frac{(\varepsilon_1-\varepsilon_0)a_1^3 + (2\varepsilon_1+\varepsilon_0)\dfrac{(\varepsilon_2-\varepsilon_1)a_2^3 + (2\varepsilon_2+\varepsilon_1)\dfrac{(\varepsilon_3-\varepsilon_2)a_3^3+\cdots}{(\varepsilon_3+2\varepsilon_2)+\cdots}}{(\varepsilon_2+2\varepsilon_1) + 2(\varepsilon_2-\varepsilon_1)a_2^{-3}\dfrac{(\varepsilon_3-\varepsilon_2)a_3^3+\cdots}{(\varepsilon_3+2\varepsilon_2)+\cdots}}}{(\varepsilon_1+2\varepsilon_0) + 2(\varepsilon_1-\varepsilon_0)a_1^{-3}\dfrac{(\varepsilon_2-\varepsilon_1)a_2^3 + (2\varepsilon_2+\varepsilon_1)\dfrac{(\varepsilon_3-\varepsilon_2)a_3^3+\cdots}{(\varepsilon_3+2\varepsilon_2)+\cdots}}{(\varepsilon_2+2\varepsilon_1) + 2(\varepsilon_2-\varepsilon_1)a_2^{-3}\dfrac{(\varepsilon_3-\varepsilon_2)a_3^3+\cdots}{(\varepsilon_3+2\varepsilon_2)+\cdots}}}, \tag{S1}$$

which makes explicit how direct symbolic expansions become increasingly cumbersome as the number of layers grows. We considered a three-layer of Au@Al_2O_3@Au, nearly spherical configuration specified in Cartesian semi-axes by $(h_1, r_1) = (0.02, 0.01999)$, $(h_2, r_2) \simeq (0.0199801, 0.0197910)$, and $(h_3, r_3) \simeq (0.0196042, 0.0195940)$ (in the same length units used to generate the spectra). In our confocal-coordinate implementation, we used the explicit surface parameters $\xi_0 = 4.1469$, $\xi_1 = 4.1369$, and $\xi_2 = 4.1269$ for the three interfaces. In Fig. S5, the solid red curve is obtained from the present

formulation (evaluated in the spherical limit), while the green dashed curve is computed from Eq. (S1), i.e. Eq. (2) of [5]. The two formulations produce indistinguishable results for Re$\{\alpha\}$ over the studied wavelength range.

S6 Conclusion

This Electronic Supplementary Material underscores the efficiency and versatility of our analytical model to calculate the optical properties of multilayered nanostructures with high proficiency and minimal computational cost using a variety of materials, extending beyond gold to include silver, aluminium, and copper. This model effectively handles diverse materials and complex geometries, highlighting its robustness and applicability across a broad range of optical applications, from plasmonic devices to photonic materials. It facilitates rapid and precise simulations, enabling the exploration and optimisation of innovative nanostructure configurations and advancing optical characterisations in the fields of nanotechnology and materials science. Additionally, the model's quick adaptability to changes in material or geometric parameters showcases its practical relevance in real-world applications, making it a foundational tool for researchers and engineers in the advancement of nano-optics and plasmonic engineering.

References

[1] Rakić, A.D., Djurišić, A.B., Elazar, J.M., Majewski, M.L.: Optical properties of metallic films for vertical-cavity optoelectronic devices. Applied Optics **37**(22), 5271–5283 (1998)

[2] Prodan, E., Radloff, C., Halas, N.J., Nordlander, P.: A hybridization model for the plasmon response of complex nanostructures. Science **302**(5644), 419–422 (2003)

[3] Prodan, E., Nordlander, P.: Plasmon hybridization in spherical nanoparticles. The Journal of Chemical Physics **120**(11), 5444–5454 (2004)

[4] Nordlander, P., Prodan, E.: Plasmon hybridization in nanoparticles near metallic surfaces. Nano Letters **4**(11), 2209–2213 (2004)

[5] Sihvola, A., Lindell, I.V.: Polarizability and effective permittivity of layered and continuously inhomogeneous dielectric ellipsoids. Journal of Electromagnetic Waves and Applications **4**(1), 1–26 (1990) https://doi.org/10.1163/156939390X00429

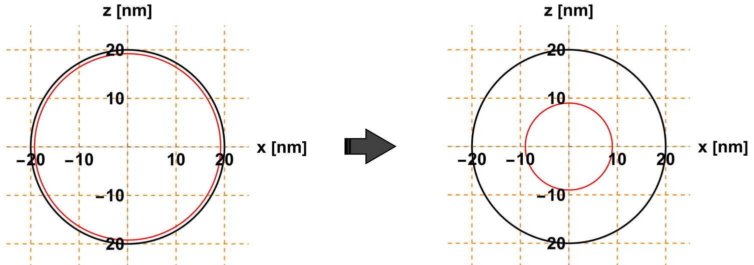

(a) Geometrical configurations of 2-layer spherical system

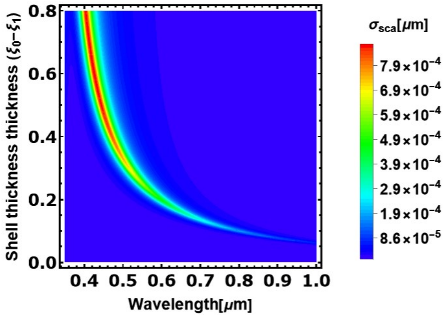

(b) Scattering cross-section for $Al_2O_3@Ag$

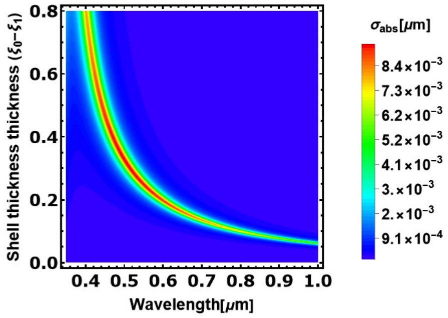

(c) Absorption cross-section for $Al_2O_3@Ag$

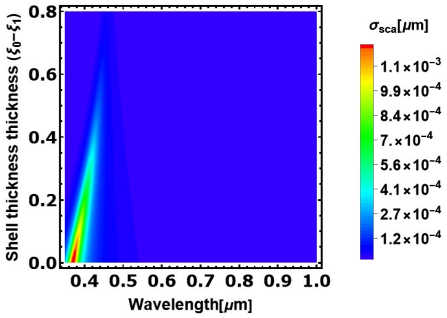

(d) Scattering cross-section for $Ag@Al_2O_3$

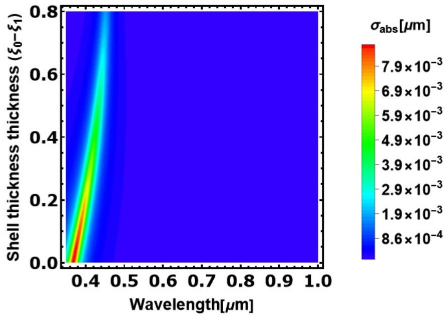

(e) Absorption cross-section for $Ag@Al_2O_3$

Fig. S1: Density plots of the optical properties for spherical core-shell nanostructures as a function of shell thickness and wavelength. (a) Illustration of the geometrical configuration used in the study. (b) and (c) depict the scattering (σ_{sca}) and absorption (σ_{abs}) cross-sections for Al_2O_3@Ag core-shell spheres, respectively. (d) and (e) present the corresponding properties for Ag@Al_2O_3 core-shell spheres. The colour bars indicate variations in magnitudes, highlighting the sensitivity of metallic shell nanostructures to changes in their geometrical configuration when the core and shell materials are interchanged.

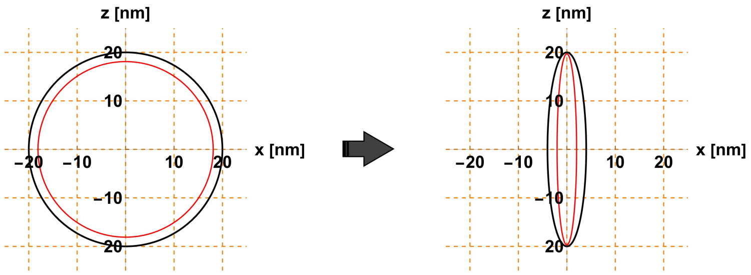

(a) Geometry configurations of 2-layer spheroidal system

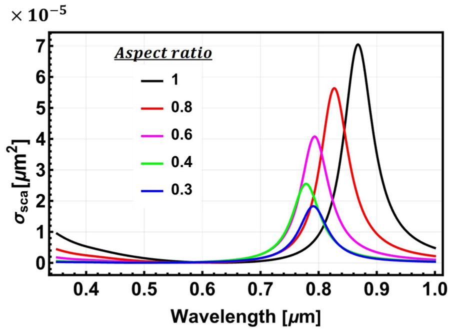

(b) Scattering cross-section for $Al_2O_3@Ag$

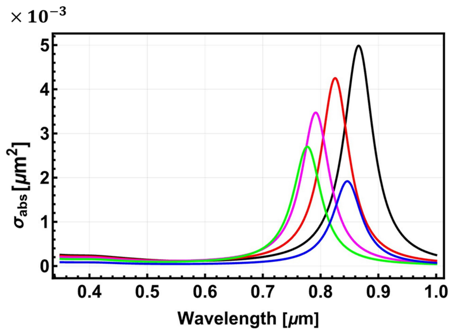

(c) Absorption cross-section for $Al_2O_3@Ag$

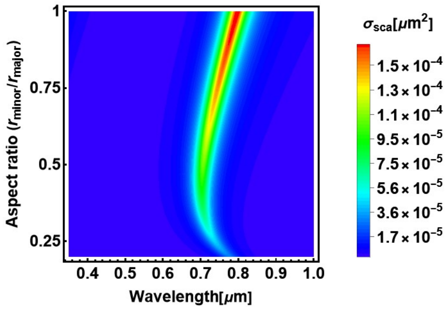

(d) Scattering cross-section for $Ag@Al_2O_3$

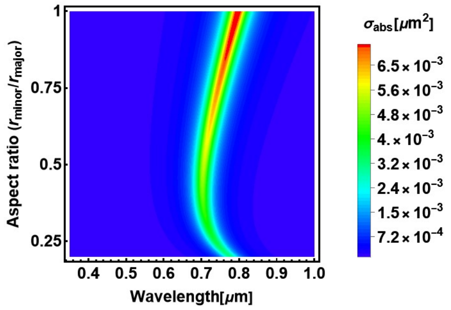

(e) Absorption cross-section for $Ag@Al_2O_3$

Fig. S2: Density plots of the optical properties for spheroidal core-shell nanostructures as a function of shell thickness and wavelength. (a) Illustration of the geometrical configuration used in the study. (b) and (c) depict the scattering (σ_{sca}) and absorption (σ_{abs}) cross-sections, respectively, for Al_2O_3@Ag core-shell nanospheroids. The colour bars indicate variations in magnitudes, highlighting the competing effects of spheroid eccentricity and shell thickness in configurations with a metallic shell. (d) and (e) present the corresponding properties for Ag@Al_2O_3 core-shell nanospheroids. The colour bars here highlight the sensitivity of the resonance red-shift in configurations with the metallic core.

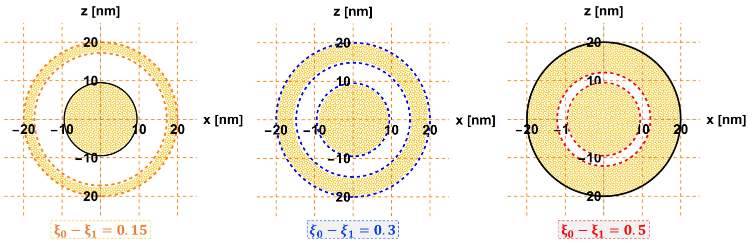

(a) Geometry configurations of 3-layer spherical system

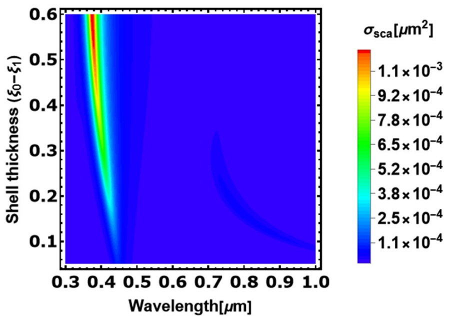

(b) Scattering cross-section for Ag@Al_2O_3@Ag

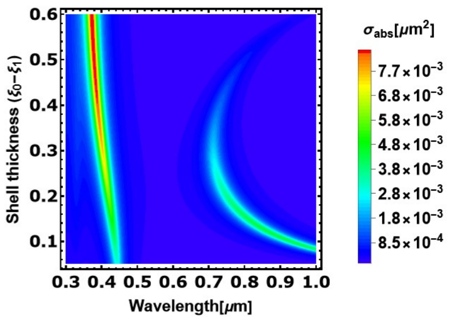

(c) Absorption cross-section for Ag@Al_2O_3@Ag

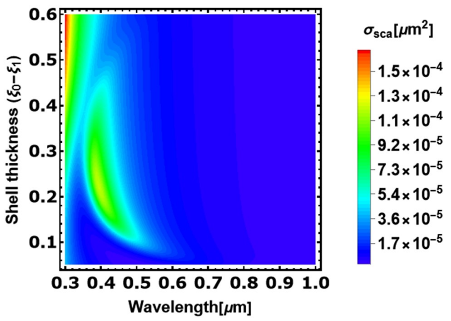

(d) Scattering cross-section for Al@Al_2O_3@Al

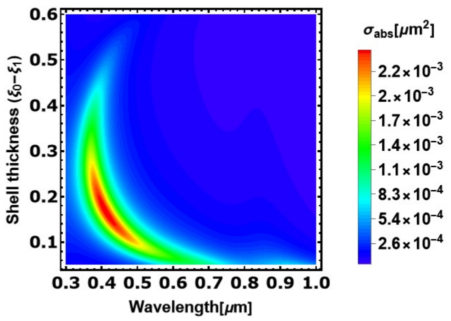

(e) Absorption cross-section for Al@Al_2O_3@Al

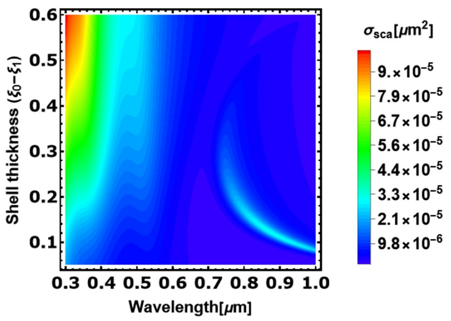

(f) Scattering cross-section for Cu@Al_2O_3@Cu

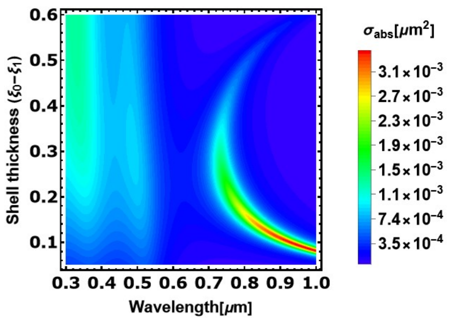

(g) Absorption cross-section for Cu@Al_2O_3@Cu

Fig. S3: Density plots of the optical properties for 3-layer spherical nanostructures as a function of shell thickness and wavelength.(a) Geometrical configurations at three different intermediate layer thicknesses: $\xi_0 - \xi_1 = 0.15$, 0.3, and 0.5. (b) and (c) Density plots of the scattering (σ_{sca}) and absorption (σ_{abs}) cross-section spectra, respectively, for Ag@Al_2O_3@Ag. (d) and (e) Density plots of the scattering and absorption cross-section spectra, respectively, for Al@Al_2O_3@Al. (f) and (g) Density plots of the scattering (σ_{sca}) and absorption (σ_{abs}) cross-section spectra, respectively for Cu@Al_2O_3@Cu.

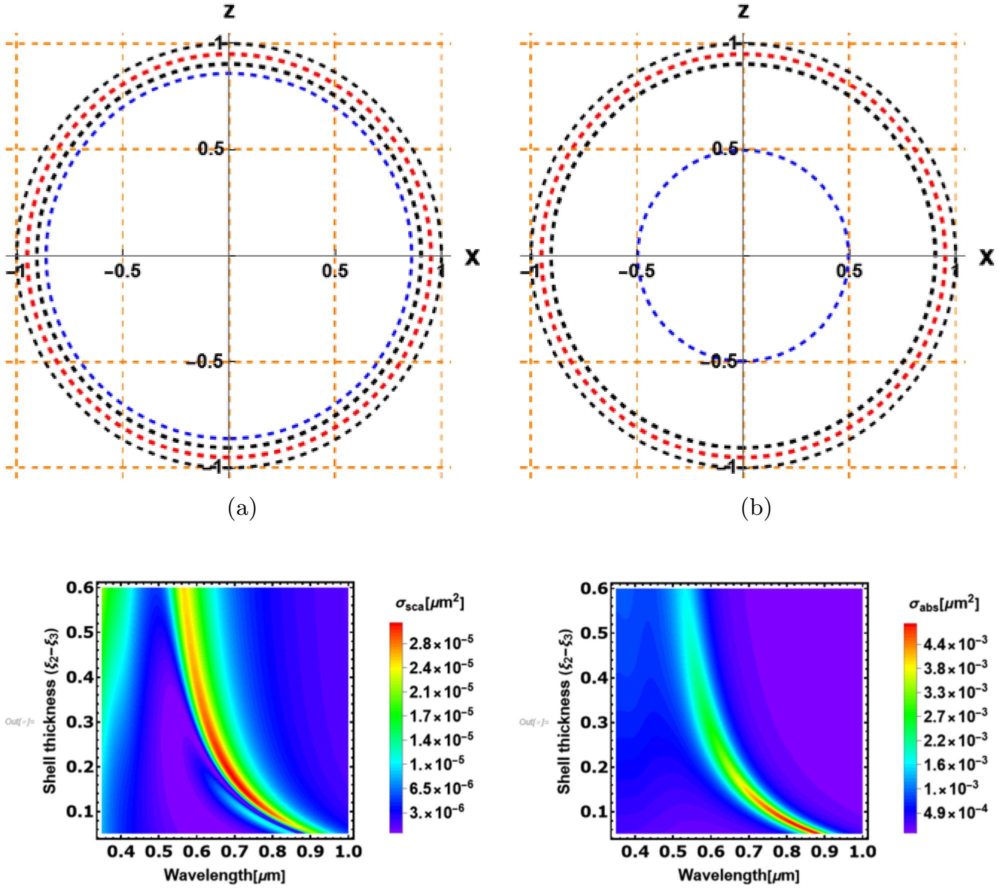

(a) (b) (c) (d)

Fig. S4: Four-layer validation case with an innermost-interface sweep. (a) Initial configuration ($\xi_3 = 7.10433$). (b) Final configuration ($\xi_3 = 6.55433$). (c) and (d) Density plots of the scattering and absorption cross-section spectra, respectively, for Au@Al_2O_3@Au@Al_2O_3. The absorption map shows stronger response for thinner effective shells (smaller $\xi_2 - \xi_3$), while the scattering map exhibits an optimised band at intermediate thickness values (roughly $\xi_2 - \xi_3 \sim 0.1 - 0.4$) depending on wavelength.

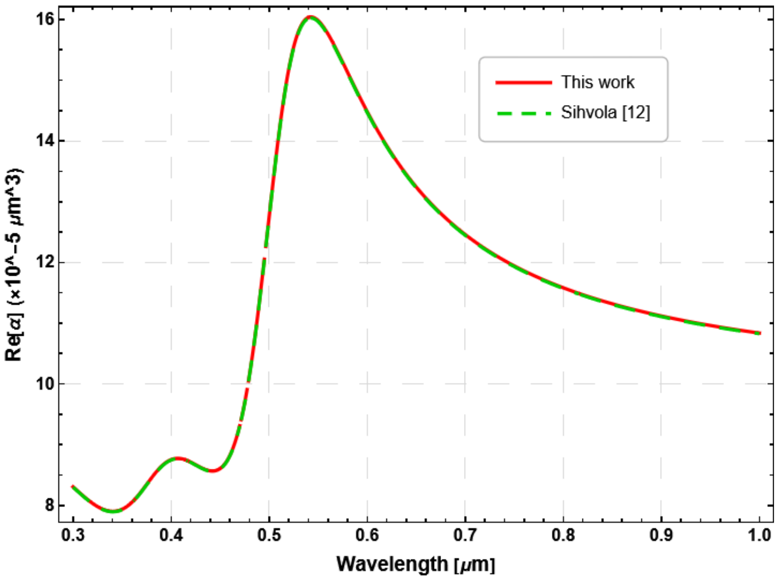

Fig. S5: Validation of the layered-sphere (dipole) limit: comparison of Re$\{\alpha\}$ of Au@Al_2O_3@Au configuration computed using the present model (solid red) against Eq. (2) in Sihvola and Lindell [5] (green dashed) for a three-layer, nearly spherical configuration.
